# Activation of a *Neospora caninum* EGFR-Like Kinase Facilitates Intracellular Parasite Proliferation

**DOI:** 10.3389/fmicb.2017.01980

**Published:** 2017-10-12

**Authors:** Xiaoxia Jin, Guojiang Li, Xichen Zhang, Pengtao Gong, Yanhui Yu, Jianhua Li

**Affiliations:** ^1^Key Laboratory of Zoonosis, Ministry of Education, College of Veterinary Medicine, Jilin University, Changchun, China; ^2^Jilin Agricultural Science and Technology University, Jilin, China

**Keywords:** *N. caninum*, EGFR, AG1478, PKC activity, parasite proliferation

## Abstract

The Apicomplexan parasite *Neospora caninum*, an obligate intracellular protozoan, causes serious diseases in a number of mammalian species, especially in cattle. Infection with *N. caninum* is associated with abortions in both dairy and beef cattle worldwide which have a major economic impact on the cattle industry. However, the mechanism by which *N. caninum* proliferates within host cells is poorly understood. Epidermal growth factor receptor (EGFR) is a protein kinase ubiquitously expressed, present on cell surfaces in numerous species, which has been confirmed to be essential in signal transduction involved in cell growth, proliferation, survival, and many other intracellular processes. However, the presence of EGFR in *N. caninum* and its role in *N. caninum* proliferation remain unclear. In the present study, we identified a putative EGFR-like kinase in *N. caninum*, which could be activated in tachyzoites by infection or treatment with rNcMIC3 [containing four epidermal growth factor (EGF) domains] or human EGF. Blockade of EGFR-like in tachyzoites by AG1478 significantly reduced parasite proliferation in host cells. Our data suggested that the activation of tachyzoite EGFR-like might facilitate the intracellular proliferation of *N. caninum*.

## Introduction

*Neospora caninum*, classified in the phylum Apicomplexa (Dubey et al., 2002), infects a variety of mammalian species with a worldwide distribution which results in severe protozoosis–Neosporosis (Dubey, 2003). Infection with *N. caninum* mainly leads to abortions and stillbirths in pregnant herds and neuromuscular disorders in newborns which cause huge economic losses in the animal husbandry, especially in the cattle production. *N. caninum* is regarded as the main cause of abortions in dairy cattle throughout the world (Reichel and Ellis, 2008; Dubey and Schares, 2011). Like most of Apicomplexan protozoa, *N. caninum* is an obligate intracellular parasite. The process of host cells infection by *N. caninum* includes adhesion, invasion, parasitophorous vacuole formation, and polypide growth. After invasion, replication of *N. caninum* in host cells leads to cell lysis, which results in the release of parasites to invade neighboring cells to repeat the life cycle and establish infection (Innes et al., 2005; Dubey et al., 2007). However, the mechanism by which *N. caninum* proliferates within host cells is not fully elucidated.

The epidermal growth factor receptor (EGFR) is a transmembrane receptor with intrinsic tyrosine kinase (TK) activity belonging to the HER/ErbB protein family, which including HER1 (erbB1, EGFR), HER2 (erbB2, NEU), HER3 (erbB3), and HER4 (erbB4). EGFR, widely distributed on the cell membrane among many mammalian species, is a major signal transduction molecule involved in various cellular activities. EGFR signaling pathway plays crucial roles in the regulation of many physiological and pathological processes, such as cell growth, differentiation, migration, apoptosis, and proliferation. EGFR is activated by ligand binding, including epidermal growth factor (EGF) and transforming growth factor alpha. Ligand binding induces receptor homodimerization or heterodimerization with other ErbB/HER members which in turn causes autophosphorylation of EGFR tyrosine residues, resulting in the activation of downstream signal transduction through MAPK, PI3K/Akt, and SAPK pathways (Yang et al., 2017). It has been reported that host EGF binds to EGFR on *Trypanosoma cruzi* amastigotes to induce signal transduction events and cell proliferation (Ghansah et al., 2002). Inhibition of EGFR TKs blocked the development of *Toxoplasma gondii*, *Sarcocystis neurona*, *Cryptosporidium parvum*, and *N. caninum* (Gargala et al., 2005; Yang et al., 2014). However, so far EGFR homologs or EGFR-like mediated signal transduction in Apicomplexan parasites have not been reported.

The aim of the present study is to investigate whether EGFR homologs exist in *N. caninum* and its potential function on parasite proliferation in host cells. We found that *N. caninum* possessed a putative EGFR-like protein in tachyzoites which could be activated by *N. caninum* infection or by recombinant NcMIC3 and human EGF (hEGF). The activation of the putative EGFR-like protein could be inhibited by EGFR-specific inhibitor-AG1478. Tachyzoite EGFR-like presented rNcMIC3- and hEGF-dependent protein kinase C (PKC) activities which could be inhibited by AG1478. In addition, inhibition of tachyzoites EGFR-TK by AG1478 resulted in decreased parasite proliferation within host cells.

## Materials and Methods

### Reagents and Antibodies

Specific EGFR and phospho-EGFR (Tyr1068) rabbit monoclonal antibodies (mAb) were from Cell Signaling Technology, Inc. (Beverly, MA, United States). AG1478 were from Selleck Chemical., Co. (Houston, TX, United States). Beta-actin mouse mAb was from Proteintech (West Grove, PA, United States). Cy3- or FITC-conjugated goat anti-rabbit or anti-mouse IgG, peroxidase-conjugated goat anti-rabbit and goat anti-mouse IgG were all from Proteintech. Recombinant hEGF was from Sigma Chemical., Co. (St. Louis, MO, United States). Prokaryotic recombinant protein NcMIC3 (rNcMIC3) and mouse antiserum specific for NcSAG1 was produced in our Laboratory. Full-length gene of *N. caninum* MIC3 was subcloned into the pET-28a (+) vector (TaKaRa, Japan) containing the His-tag, and the recombination pET-28a-NcMIC3 contains four EGF sequence repeats.

### Cell Culture

HEK-293 cells (ATCCCRL-1573) and Vero cells (ATCCCCL-81) were cultured in DMEM supplemented with 10% heat-inactivated fetal bovine serum (FBS) and antibiotic–antimycotic reagents (all from Gibco BRL, Rockville, MD, United States). One day before treatment, the culture medium was changed to DMEM with 2% FBS.

### *Neospora caninum* Culture and Purification

Tachyzoites of *N. caninum*-1 strain (ATCC 50843) were restored at our laboratory. Vero cells were infected with tachyzoites of Nc-1 and cultured at 37°C and 5% CO_2_ for 3–5 days in DMEM/F12 supplemented with 2% heat-inactivated FBS and antibiotic–antimycotic reagents. After spontaneous host cell rupture, parasites and cell debris were washed in cold DMEM/F12 without FBS and harvested by centrifugation at 850 × *g* at 4°C for 10 min. After centrifugation, the pellet was resuspended in cold DMEM/F12 and passed through a 26-gauge needle (Millipore, Billerica, MA, United States) to further break the cells. The obtained mixture was slowly added onto 40% percoll solution (GE Healthcare, United States) in DMEM/F12 without FBS and separated by centrifugation at 850 × *g* in a horizontal centrifuge for 30 min. The fraction containing tachyzoites at the bottom of the tube was collected, resuspended in DMEM/F12 without FBS and centrifuged at 850 × *g* at 4°C for 10 min. The final pellet was purified tachyzoites.

### Immunofluorescence

HEK-293 cells were cultured in 24-well plates containing glass slides and serum-starved overnight. Serum-starved Nc-1 tachyzoites were inoculated into the cells or not at a MOI of 10 for 10 min. For parasite immunofluorescence, purified Nc-1 tachyzoites were fixed on coverslips coated with polylysine. Coverslips were rinsed in 1× PBS with 0.05% Tween-20. Monolayers were fixed with 4% paraformaldehyde for 15 min, washed three times in PBST and permeabilized in cold 0.25% Triton X-100 (Life Technologies Corporation, CA, United States) for 10 min. After PBST washing, coverslips were blocked in 5% w/v bovine serum albumin (BSA) for 1 h at 37°C. Slides were incubated in the mAb against phospho-tyrosine 1068 EGFR and EGFR (1/1,000) or antiserum against NcSAG1 (1/100) in PBST containing 3% BSA for 1 h at 37°C. After washing three times in PBST, slides were incubated with the corresponding secondary antibody conjugated to Cy3 or FITC (1/500) in PBST containing 3% BSA for 45 min in the dark. Slides were washed three times in PBST and counter stained with DAPI (1/1,000; GeneCopoeia, United States) for 5 min. After washing, monolayers were then observed using a FV1000 confocal microscopy (Olympus Co., Japan). Specificity of staining was determined by incubating monolayers with secondary antibody only.

### Immunoblotting

Nc-1 tachyzoites were prepared as above. For treatment with EGFs, 2 × 10^6^ tachyzoites were serum-starved overnight and subjected to treatments with 25–400 ng/ml hEGF or vehicles for 10 min, and with 0 or 100 ng/ml rNcMIC3 for 5–120 min, respectively. Some sets of tachyzoites (2 × 10^6^) were incubated with 100 ng/ml rNcMIC3 or 50 ng/ml hEGF for 0, 10, 30, and 60 min in the presence of AG1478 (20 μM) or DMSO. Before and after treatments, parasite pellets were collected, rinsed with cold PBS for three times, and gently sonicated on ice for 15 s (amplitude 30, 5 s for 10 s; Sonics and Materials Inc., United States) in RIPA lysis buffer supplemented with protease and phosphatase inhibitors and PMSF (all from Sangon Biotech Co., Shanghai, China) and then lysed on ice. After mixing with 5× loading buffer, protein extracts were boiled for 5 min and centrifuged at 10,000 × *g* for 5 min at 4°C. The supernatants were then subjected to electrophoresis on 10% SDS-PAGE gels (Bio-Rad Laboratories, Inc., United States) in the same amount and transferred to PVDF membrane (Pall Life Sciences, United States) by electroblotting (Bio-Rad). Membranes were blocked for 1 h in 5% (w/v) skim milk in TBST. The expression of *N. caninum* EGFR was detected using antibody to total EGFR (170 kDa, 1/1,000) or phospho-tyrosine 1068 EGFR (170 kDa, 1/1,000) in TBST overnight at 4°C, followed by four times washing and incubation with horseradish peroxidase-conjugated affinipure goat anti-rabbit IgG (1/2,500) in TBST for 1 h at room temperature. Protein bands were visualized by using an enhanced chemiluminescence kit (Proteintech Group Inc., United States) and detected using ChemiScope series 5300 (Clinx Science Instruments Co., Ltd., Shanghai, China) according to the manufacturer’s instructions. Beta-actin and NcSAG1 was used as the loading control. Intensities of phospho-EGFR were calculated using ImageJ (NIH) and normalized against total EGFR.

### PKC Activity Assay

To determine whether rNcMIC3 or hEGF could induce PKC activity in tachyzoites, a non-radioactive PKC assay kit (Calbiochem, La Jolla, CA, United States) was used. Serum-starved tachyzoites were prepared and subjected to different treatments. One set of tachyzoites (1 × 10^7^) were incubated with rNcMIC3 at concentrations of 0.01–1 μg/ml or with vehicles at 37°C for 10 min. One set of tachyzoites (1 × 10^7^) were incubated with 0 or 0.05 μg/ml hEGF for 10–60 min. To test for activity inhibition, tachyzoites (1 × 10^7^) were incubated with or without the EGFR inhibitor AG1478 (20 μM) for 2 h, and then stimulated with 0.1 μg/ml rNcMIC3 or 0.05 μg/ml hEGF for 10 min. After treatments, tachyzoites pellets were collected, rinsed with HEPES buffer, and lysates were obtained as described above. The lysates were centrifuged at 10,000 × *g* for 15 min at 4°C and supernatants were used for protein-kinase activity analysis. PKC activity was measured with ELISA according to the manufacturer’s instructions. Samples were applied in triplicates in 96-well plates and read at 450 nm on a Powerwave 200 spectrophotometer (Bio-Tek, Winooski, VT, United States). Relative PKC activity was calculated by dividing PKC activity induced by EGF by the PKC activity of its respective mock-treated control.

### Proliferation of *N. caninum*

HEK-293 cells were cultured in 24-well plates containing glass coverslips prior to inoculation with freshly purified Nc-1 tachyzoites at a MOI of 10 in the presence of AG1478 or DMSO with the concentration of 20 μM. For pretreatment on parasites only, tachyzoites were pretreated with DMSO or AG1478 (20 μM), then inoculated to HEK-293 cells after rinse with PBS. At 2 h, the coverslips were rinsed with PBS to remove free tachyzoites. After 24 h of incubation, the coverslips were rinsed three times with PBS and cells were collected and genome were obtained using a Genome DNA Extraction Kit (Sangon Biotech Co., China). Then the DNA was adjusted to 20 ng/μl and the parasite burden was determined by real-time fluorescent quantitative PCR. The Nc-5 sequence of *N. caninum* was detected using a BioEasy SYBR Green I Real Time PCR Kit (Roche, United States) following the manufacturer’s instructions with the primer pairs (forward primer, 5′-TCCCTC GGTTCACCCGTTCACACAC-3′, reverse primer, 5′-CACGTATCCCACCTC TCACCGCTACCA-3′). Cell GAPDH were used as the control and the relative numbers of *N. caninum* in cells were calculated by SPSS. In addition, some sets of monolayers were fixed with cold methyl alcohol and stained with acridine orange (Life Technologies, United States), which were observed using a FV1000 confocal microscopy (Olympus Co., Japan). The number of tachyzoites per 100 cells and the numbers of tachyzoites in vacuoles were determined by counting at ×1,000 magnification. Experimental groups had triplicate samples and at least 100-200 cells or 100 vacuoles per sample were counted.

### Statistical Analysis

Results were obtained from triplicate values in each independent experiment and analyzed for statistical significance using Student’s *t*-test and one-way ANOVA by SPSS 19.0 software. Results are expressed as the mean ± standard errors. Differences were considered statistically significant when *p*-values were <0.05 (^∗^*p* < 0.05 and ^∗∗^*p* < 0.01).

## Results

### *Neospora caninum* Infection Induced EGFR-Like Activation in Tachyzoites

**FIGURE 1:**
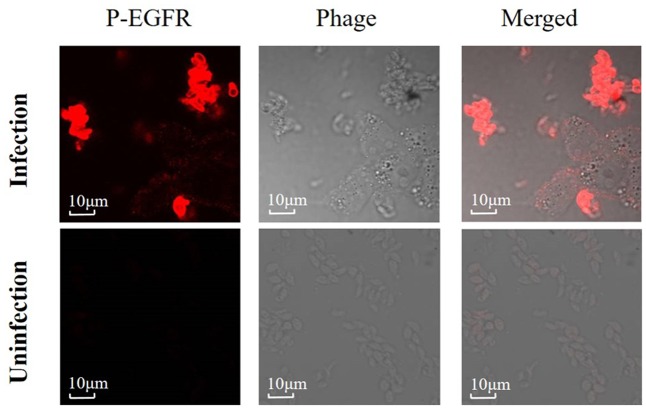
*Neospora caninum* infection induces EGFR-like activation in tachyzoites. Purified *N. caninum* tachyzoites (Nc-1) were inoculated into HEK-293 cells or not at a MOI of 10, then phosphorylation of EGFR (Tyr1068) were detected at 10 min by immunofluorescence. Results are representative of three independent experiments.

To examine whether *N. caninum* could induce phosphorylation of EGFR on the cell surface, purified tachyzoites were inoculated to HEK-293 cells. Immunofluorescence staining with tyrosine residue 1068 specific phosphor antibody suggested that not only cell EGFR was phosphorylated; *N. caninum* infection also induced activation of EGFR-like on tachyzoites themselves (**Figure 1**). Phospho-EGFR-specific fluorescence could be observed as early as 10 min after infection, while phospho-EGFR (Tyr1068) expression could not be observed in tachyzoites which were not inoculated into cells, as shown in **Figure 1**. Thus, *N. caninum* infection caused rapid EGFR-like activation in tachyzoites.

### Identification of Putative EGFR-Like in *N. caninum* Tachyzoites

**FIGURE 2:**
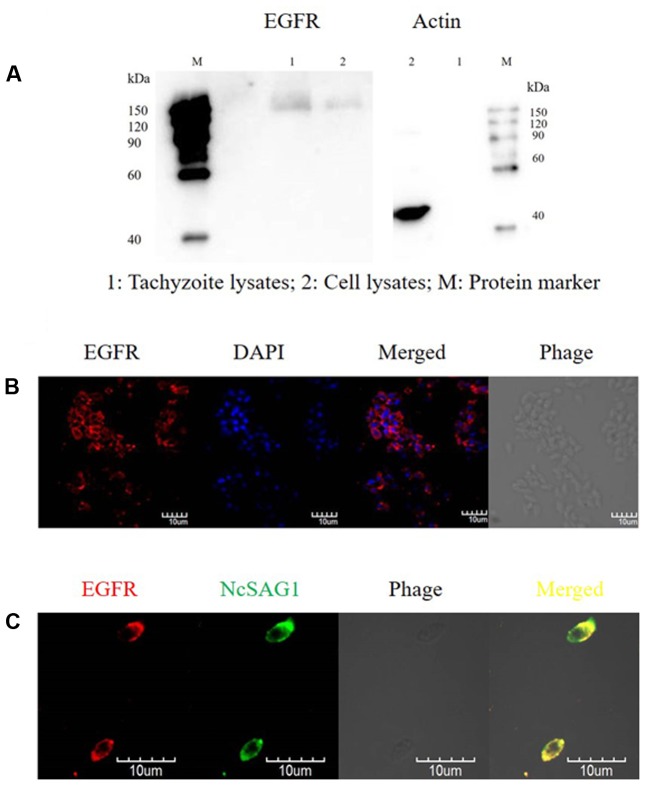
Identification of putative EGFR-like in *N. caninum* tachyzoites. **(A)** *Neospora caninum* tachyzoites (2 × 10^6^) and HEK-293 cell lysates were subject to immunoblot to examine the expression of EGFR using mAb specifically against EGFR (170 kDa, 1:1,000) and β-actin (45 kDa, 1:2,000), respectively; **(B)** 1 × 10^6^ Nc-1 tachyzoites were purified and protein expression of *N. caninum* EGFR was detected by immunofluorescence; **(C)** purified Nc-1 tachyzoites (1 × 10^6^) were subjected to IFA with antibodies against EGFR and NcSAG1. Tachyzoites were observed using a FV1000 confocal microscopy. Results are representative of three independent experiments.

In order to determine the expression of parasite EGFR, protein extracts from *N. caninum* tachyzoites and HEK-293 cells were subject to immunoblot using mAb against EGFR and β-actin, respectively. An EGFR-specific protein band with a molecular weight of 170 kDa was shown in both tachyzoite and HEK-293 cell lysates as shown in the left panel of **Figure 2A**. Moreover, the β-actin protein band (45 kDa) was present in HEK-293 cell lysates while not in tachyzoite lysates, suggesting that tachyzoite extracts were not contaminated with cell proteins as shown in the right panel of **Figure 2A**. Meanwhile, the results from immunofluorescence staining using the antibody specifically against EGFR also suggested the expression of EGFR-like in *N. caninum* tachyzoites (**Figure 2B**). In addition, co-localization of the EGFR-like with SAG1 in *N. caninum* by IFA indicated that *N. caninum* EGFR-like located on the tachyzoite surfaces (**Figure 2C**).

### EGF-Induced PKC Activity in *N. caninum* Tachyzoites

**FIGURE 3:**
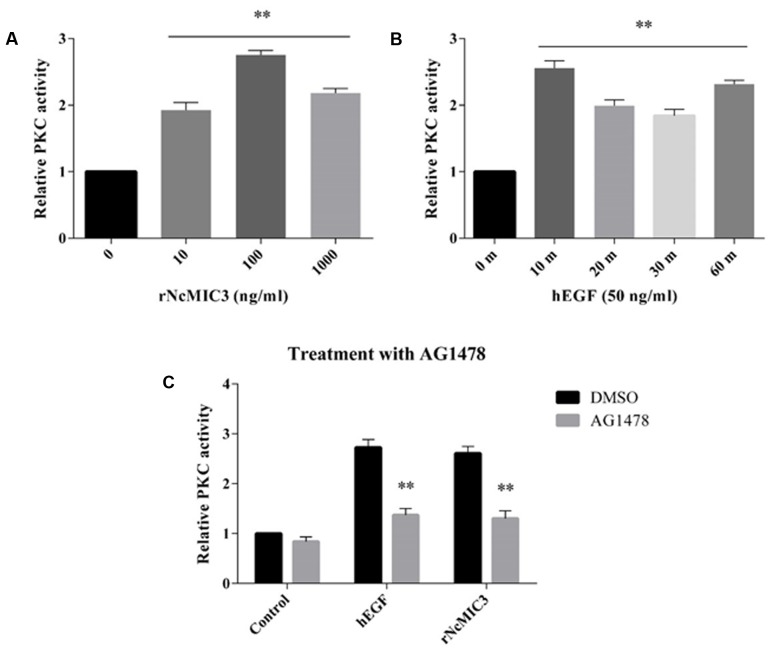
EGF-induced PKC activity in *N. caninum* tachyzoites. **(A)** Nc-1 tachyzoites (1 × 10^7^) were incubated with 0, 10, 100, or 1,000 ng/ml rNcMIC3 at 37°C for 10 min; **(B)** 1 × 10^7^ tachyzoites were incubated with 0 or 50 ng/ml hEGF for 10–60 min at 37°C; **(C)** to test the inhibition effect by EGFR kinase inhibitor (AG1478), tachyzoites (1 × 10^7^) were incubated with 20 μM AG1478 or DMSO, then stimulated with 100 ng/ml rNcMIC3 or 50 ng/ml hEGF for 10 min at 37°C. Tachyzoite lysates were obtained for PKC activity measure by ELISA using a non-radioactive PKC assay kit. Samples were applied in triplicates. Results represent the mean of three independent experiments ± SD. ^∗^*p* < 0.05; ^∗∗^*p* < 0.01.

Next, the ability of EGFs to induce PKC activities in tachyzoites was investigated. PKC activities in tachyzoites before or after stimulation with rNcMIC3 or hEGF were measured. The results showed that after treatment with the recombinant proteins, the PKC activities in tachyzoites were significantly increased in a concentration and time dependent manner. When incubated with rNcMIC3 (**Figure 3A**, *p* < 0.01) at 100 ng/ml or hEGF (**Figure 3B**, *p* < 0.01) at 50 ng/ml for 10 min, the PKC activity peaked and increased by 50.7 and 48.8% compared to control, respectively, while these increases were both effectively inhibited when treatment with 20 μM EGFR-inhibitor AG1478 (**Figure 3C**, *p* < 0.01). The results suggested that the EGF-dependent PKC activity was present in *N. caninum* tachyzoites.

### RNcMIC3 and hEGF Induced the Activation of Tachyzoite EGFR-Like

**FIGURE 4:**
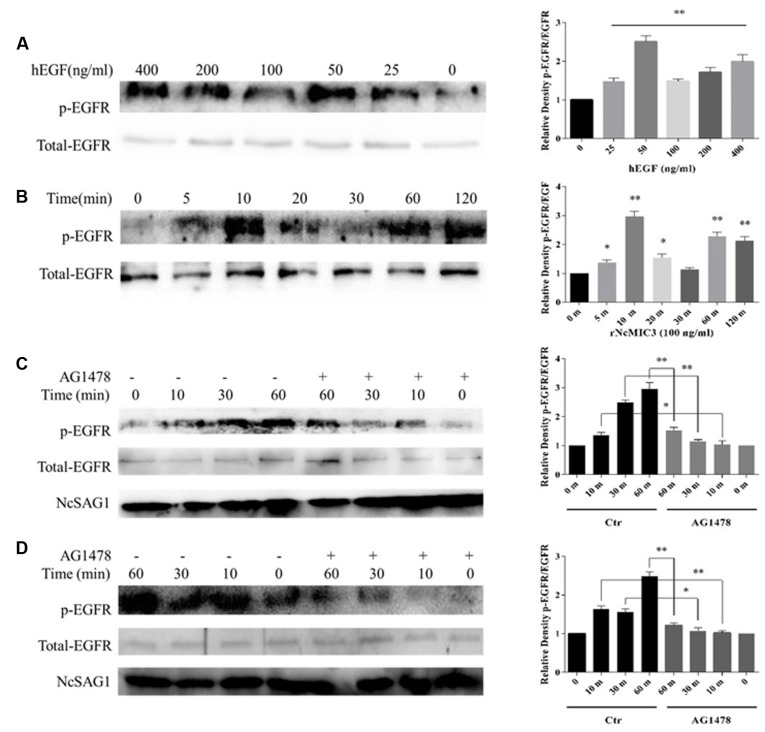
rNcMIC3 and hEGF induce the activation of tachyzoite EGFR-like. **(A)** Nc-1 tachyzoites (2 × 10^6^) were incubated with hEGF at different concentrations (0–400 ng/ml) for 10 min at 37°C; **(B)** 2 × 10^6^ tachyzoites were incubated with 0 or 100 ng/ml rNcMIC3 for 5–120 min; **(C)** 2 × 10^6^ tachyzoites were incubated with 0 or 50 ng/ml hEGF for 10, 30, and 60 min in the presence of AG1478 (20 μM) or DMSO; **(D)** 2 × 10^6^ tachyzoites were incubated with 0 or 100 ng/ml rNcMIC3 for 10, 30, and 60 min in the presence of AG1478 (20 μM) or DMSO. After treatments, tachyzoite lyses were obtained for detection of phospho-EGFR (Tyr1068, 170 kDa) and EGFR (170 kDa) by western blot. NcSAG1 was used as the loading control. EGFR phosphorylation levels were calculated after normalization to the amount of total EGFR. Bars represent the mean ± SD of three independent experiments. ^∗^*p* < 0.05; ^∗∗^*p* < 0.01.

To examine whether *N. caninum* MIC3 which contains four EGF repeats and hEGF were involved in the activation of parasite EGFR-like, purified tachyzoites were treated with or without rNcMIC3 or hEGF with or without the presence of AG1478. Immunoblot results suggested that phosphorylation of tachyzoites EGFR-like (170 kDa) increased with recombinant hEGF and NcMIC3 in a dose- and time-dependent manner, respectively, compared to control. EGFs-induced phosphorylation of parasite EGFR-like peaked at 50 ng/ml of hEGF (**Figure 4A**, *p* < 0.01) or at 100 ng/ml of rNcMIC3 (**Figure 4B**, *p* < 0.01) both for 10 min. Furthermore, results from co-incubation of 50 ng/ml hEGF (**Figure 4C**, *p* < 0.05) or 100 ng/ml rNcMIC3 (**Figure 4D**, *p* < 0.05) with 20 μM AG1478 for 10–60 min showed that the phosphorylation levels of tachyzoites EGFR-like induced by the recombinant proteins were significantly decreased at every time point. These results showed that rNcMIC3 and hEGF promoted the activation of tachyzoite EGFR-like.

### Activated Tachyzoite EGFR-Like Promoted Intracellular *N. caninum* Proliferation

**FIGURE 5:**
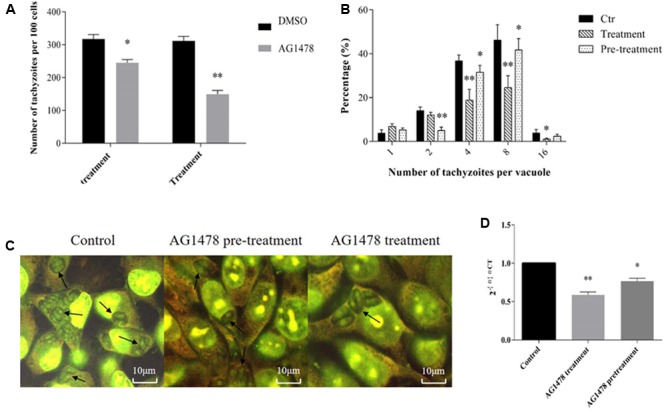
Activated tachyzoite EGFR-like promotes *N. caninum* proliferation. For treatment, HEK-293 cells were inoculation with Nc-1 tachyzoites at a MOI of 10 in the presence of AG1478 (20 μM) or DMSO (Ctr); for pretreatment, tachyzoites were pretreated with DMSO or AG1478 (20 μM) for 2 h, followed by inoculating tachyzoites to HEK-293 cells after rinse with PBS. Parasite development in HEK-293 cells at 24 h were detected by acridine orange staining, which were observed using a FV1000 confocal microscopy. The number of tachyzoites/100 cells **(A)** and the numbers of tachyzoites in vacuoles **(B)** were determined by counting at ×1,000 magnification. Experimental groups had triplicate samples and at least 100–200 cells or 100 vacuoles per sample were counted; **(C)** representative pictures from acridine orange staining; **(D)** HEK-293 cells were inoculated with tachyzoites at a MOI of 10 before (Ctr) or after treatment and pretreatment with AG1478 (20 μM). *N. caninum* burden were detected by quantitative real-time PCR with Nc-5-specific sequence for *N. caninum*. The relative numbers of *N. caninum* were calculated by SPSS. Data are expressed as the mean of three independent experiments ± SD. ^∗^*p* < 0.05; ^∗∗^*p* < 0.01.

Finally, we explored the role of tachyzoite EGFR-like activation in parasite proliferation in host cells. The results from acridine orange staining indicated that treatment or pretreatment with specific EGFR-inhibitor AG1478 (20 μM, which was close to the highest IC50 and exhibited no toxicity to cells) obviously decreased the ability of the tachyzoites to grow within vacuoles in HEK-293 cells. AG1478 treatment decreased not only the number of tachyzoites per 100 cells by 52.9% (**Figures 5A,C**, *p* < 0.01), but also the number of tachyzoites per vacuole (**Figures 5B,C**, *p* < 0.01) at 24 h. AG1478 pretreatment on tachyzoites only induced a small but significant reduction in the number of tachyzoites per 100 cells of 21.2% and per vacuole at 24 h (**Figures 5A–C**, *p* < 0.05). In addition, the results from real-time fluorescent quantitative PCR showed that treatment or pretreatment with AG1478 significantly reduced the parasite burden in HEK-293 cells by 42.3% (*p* < 0.01) and 23.4% (*p* < 0.05) at 24 h, respectively (**Figure 5D**). These data suggested that inhibition of EGFR kinase activities both on parasites and hosts by AG1478 hindered the intracellular development of parasites, suggesting that tachyzoite EGFR-like might be involved in *N. caninum* proliferation within host cells.

## Discussion

EGFR is a receptor TK of 170 kDa belonging to the HER/ErbB protein family. EGFR signaling pathway plays critical roles in numerous cellular physiological processes. EGFR has been identified and sequenced in many animal species (Yang et al., 2017). EGFR homologs have been identified in *Drosophila*, *Caenorhabditis elegans*, *Schistosoma mansoni*, and *Trypanosoma brucei* trypomastigotes, *T. cruzi* amastigotes and *Echinococcus multilocularis* (Ghansah et al., 2002; Spiliotis et al., 2003). This study was the first to identify a putative EGFR-like kinase on Apicomplexan protozoan through Western-blot analysis and immunofluorescence staining. Furthermore, co-localization with NcSAG1 confirmed that *N. caninum* EGFR-like distributed on the surfaces of tachyzoites.

In the present study, we discovered that EGFR-like tyrosine residue of tachyzoites phosphorylated during cell infection, which could be inhibited by EGFR-inhibitor AG1478. Ligand binding to EGFR results in receptor dimerization, autophosphorylation, and activation of downstream signaling pathways. Activation of *N. caninum* EGFR might well be mediated by the binding of EGF domain containing ligand, such as host EGF or microneme proteins (MICs) that contain EGF domains. This binding induces receptor phosphorylation similar to those observed upon the binding of EGF to EGFR in mammalian cells. It has been shown that host EGF binds to EGFR on *T. cruzi* amastigotes induces the activation of protein kinases and signal transduction in amastigotes (Ghansah et al., 2002). Data from the present study suggested that both recombinant NcMIC3 and hEGF could induce the activation of parasite EGFR-like, which could be inhibited by AG1478. When exposed to rNcMIC3 or hEGF, *N. caninum* showed an EGF-dependent enhancement in PKC activity in tachyzoites in a dose- and time-dependent manners, which could also be inhibited by AG1478. These results indicated that EGFR-like TK might present in tachyzoites which could be activated by EGFs during cell invasion by the parasite.

*Neospora caninum* MICs contain EGF domains (MIC3, MIC6, and MIC8). NcMIC3 contains a lectin-like domain and four EGF repeats. NcMIC3 and TgMIC3 both have been demonstrated to be functionally involved in parasite infection (Naguleswaran et al., 2001; Meissner et al., 2002; Yang et al., 2015). It has been shown that *N. caninum* MIC3 is a secretory adhesin which interacts with Vero cell surface proteoglycan (Naguleswaran et al., 2002). *T. gondii* MIC3 binds to both the surface of the host cell and the surface of the parasite (Garcia-Réguet et al., 2000). Our results also suggested that *N. caninum* MIC3 might act on parasite EGFR-like. Additionally, tachyzoite EGFR-like might be involved in the interaction between parasites and hosts (Dissous et al., 2006).

Because of the roles of EGFR in cell growth and proliferation of mammalians, we propose the following possibility that EGFR-like in *N. caninum* would be likely to play some roles in parasite proliferation. It has been previously reported that EGFR presents on *T. cruzi* amastigotes, which binds to host EGF to induce amastigote cell proliferation and the kinase-inhibitors AG1478 and PD89059 could inhibit the EGF-mediated growth of amastigotes (Ghansah et al., 2002). In the present study, we found that blockade of tachyzoite EGFR-TK activity by AG1478, which prevents ATP from binding to tyrosine residues of EGFR, inhibited both the number of intracellular tachyzoites and the numbers of tachyzoites per vacuoles at 24 h in HEK-293 cells by real-time fluorescent quantitative PCR and acridine orange staining, although parasite proliferation was partially restored when the inhibitor was removed, suggesting a reversibility of this inhibitor on parasite kinase homologs. After pretreatment of *N. caninum* with AG1478, parasite burden within cells and the number of tachyzoites per 100 cells reduced by 21.2 and 23.4%, respectively. These data indicated that *N. caninum* EGFR-like had a significant role in parasite proliferation within host cells. It has been shown that drugs targeting EGFR-TK activity inhibit the intracellular development of Apicomplexa. Isoflavone analogs (dihydroxyisoflavone and trihydroxydeoxybenzoin derivatives) inhibit the proliferation of *S. neurona*, *C. parvum*, and *N. caninum* (Gargala et al., 2005). Gefitinib inhibits the growth of *T. gondii* in Hela cells (Yang et al., 2014). EGFR-TK might be targeted by parasite for intracellular proliferation, which therefore could be the potential target for chemotherapy.

The EGFR is a transmembrane TK. Parasite protein kinases have been suggested to play significant roles in the invasion, growth, and proliferation of parasites (Kim, 2004; Sibley, 2013). Serine protease inhibitors block invasion of host cells by *Plasmodium falciparum* (Conseil et al., 1999) and *Toxoplasma* (Alam, 2014). In Apicomplexan parasites, several kinases have been identified (Brumlik et al., 2011; Wei et al., 2013; Brown et al., 2014; Jin et al., 2017). In addition, the existence of parasite receptor kinase protein homologs similar to mammalian host cells implies that *N. caninum* EGFR might participate in host immune evasion by the parasite, which needs to be further studied.

## Conclusion

We reported here for the first time that a putative EGFR-like kinase was present in *N. caninum* tachyzoites. *N. caninum* infection activated tachyzoite EGFR-like probably by NcMICs or host EGFs. Furthermore, a specific EGFR inhibitor (AG1478) effectively reduced EGF-dependent EGFR-like activation and PKC activity in tachyzoite and decreased parasite proliferation in host cells. Further studies will be needed to precisely identify the kinase sequences of parasite EGFR and its function during parasite infection. Results from present study will be helpful in better understanding of the complicated mechanism involved in parasite proliferation within host cells of Apicomplexan *N. caninum*.

## Author Contributions

XJ, XZ, and JL conceived and designed the study. XJ and YY performed the experiments. XJ drafted the manuscript. PG analyzed the data. JL and GL critically revised the paper. All authors read and approved the final version of the manuscript.

## Conflict of Interest Statement

The authors declare that the research was conducted in the absence of any commercial or financial relationships that could be construed as a potential conflict of interest.
